# Association of anemia with cardiovascular and cancer mortality among stroke survivors: A cohort study (NHANES 1999–2018)

**DOI:** 10.1097/MD.0000000000045250

**Published:** 2025-10-17

**Authors:** Jianya Chen, Xiaoping Xie, Hui Mai, Qiufei Cheng

**Affiliations:** a Department of Neurology, Zhanjiang Central Hospital, Guangdong Medical University, Zhanjiang, Guangdong, China; b Zhanjiang Medium Medical Department School, Zhanjiang, Guangdong, China.

**Keywords:** anemia, cause-specific mortality, competing risks model, NHANES, propensity score matching, stroke survivors

## Abstract

A substantial proportion of stroke survivors have comorbid anemia, which has been associated with poor stroke outcomes. While previous studies have linked anemia to increased all-cause mortality among stroke survivors, limited research has focused on its association with cause-specific mortality. This study investigates the relationship between anemia and cause-specific mortality in stroke survivors. A total of 1561 stroke survivors were included from the NHANES 1999 to 2018 database. Restricted cubic spline (RCS) analysis was used to examine the association between hemoglobin (HGB) levels and all-cause mortality. Propensity score matching was performed at a 1:2 ratio to balance baseline characteristics. The relationships between anemia and cardiovascular, cancer-related, and other-cause mortality were assessed using competing risks models and cumulative incidence function (CIF) curves. RCS analysis revealed an L-shaped association between HGB and all-cause mortality, with a sharp increase in mortality risk below approximately 14 g/dL. In the competing risks model, anemia was significantly associated with an increased risk of cardiovascular mortality (subdistribution hazard ratio [sHR], 1.40; 95% CI: 1.02–1.90; *P* = .03), but was not significantly associated with cancer or other-cause mortality. CIF curves showed that the difference in cardiovascular mortality risk between anemic and non-anemic groups was more pronounced before 150 months of follow-up, with the difference narrowing after 200 months. Anemia is associated with an increased risk of cardiovascular mortality among stroke survivors.

## 1. Introduction

Stroke remains one of the leading causes of death and long-term disability worldwide.^[1,2]^ Despite substantial advances in acute management and secondary prevention strategies, a significant proportion of stroke survivors continue to experience poor outcomes, including increased risk of mortality.^[3–5]^ The prognosis following stroke is influenced by a variety of clinical and demographic factors, among which anemia has gained growing attention.^[6]^

Anemia is a common comorbidity among stroke patients, with approximately 20% of survivors affected.^[7]^ Compared with age-matched adults in the general population, individuals with stroke have a higher likelihood of anemia, potentially related to malnutrition, multimorbidity, iatrogenic blood loss from phlebotomy, and bleeding associated with antithrombotic therapies.^[8–12]^ However, it often receives limited attention in clinical practice, as the focus tends to be placed on antithrombotic therapy, risk factor modification, and other targeted interventions.^[13–16]^ Emerging evidence has suggested that anemia may independently contribute to worse outcomes in stroke survivors, including elevated all-cause mortality.^[17,18]^

While several studies have demonstrated an association between anemia and overall mortality in patients with stroke, few have investigated its relationship with cause-specific mortality, such as deaths due to cardiovascular disease, cancer, or other causes. Identifying the specific causes of death associated with anemia in this population is essential for improving risk stratification and guiding post-stroke management strategies. Therefore, in this study, we aimed to examine the association between anemia and cause-specific mortality among stroke survivors using data from the National Health and Nutrition Examination Survey (NHANES) 1999 to 2018. Because cardiovascular disease and cancer account for a substantial proportion of deaths after stroke – and because anemia frequently coexists with cancer and may exacerbate heart failure,^[19–21]^ we hypothesized that anemia is associated with an increased risk of cause-specific mortality, including cardiovascular and cancer-related mortality, in stroke survivors.

## 2. Methods

### 2.1. Population

**Figure 1.**
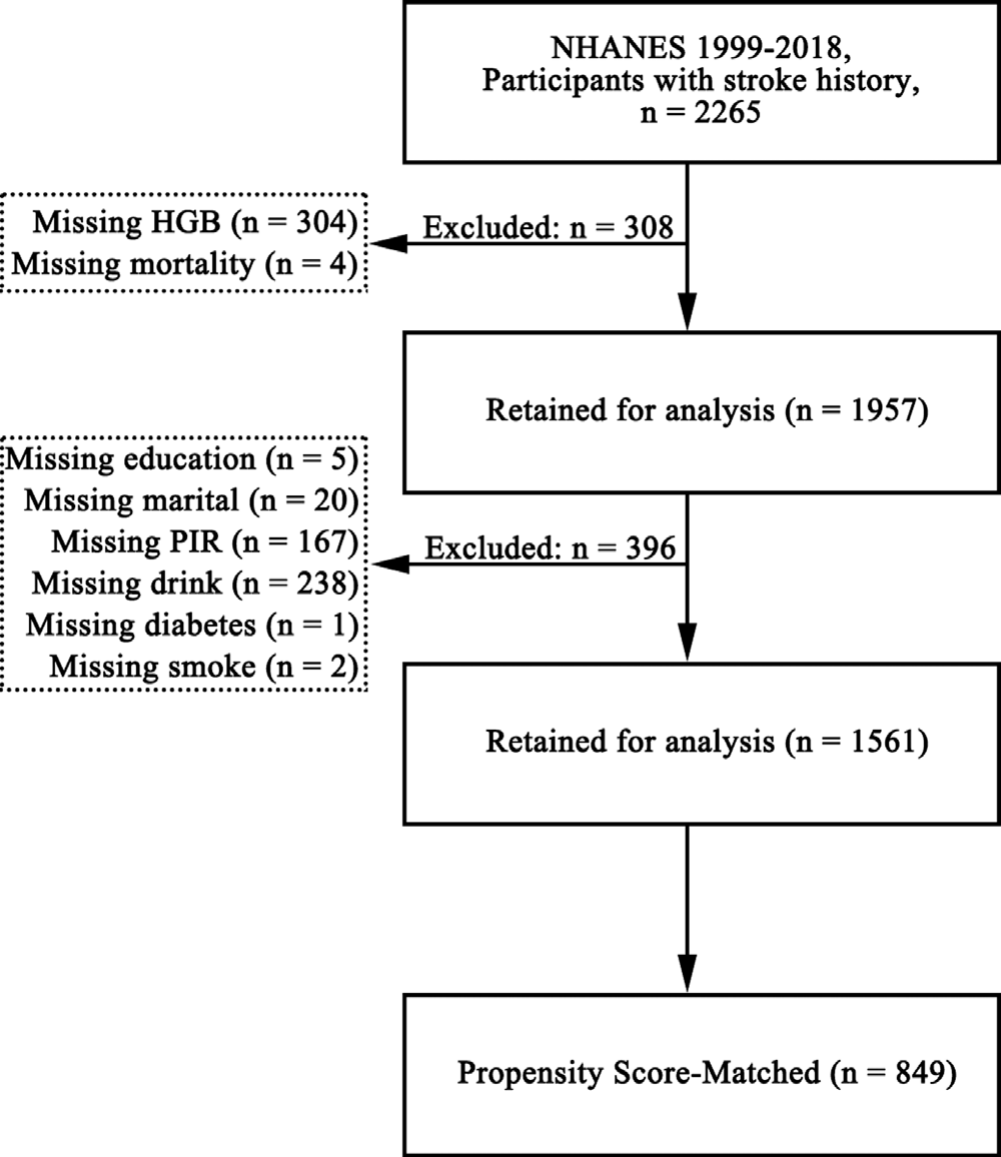
Flowchart of participants included in this study. Of 2265 NHANES 1999–2018 participants with a history of stroke, 308 with missing hemoglobin or mortality linkage and 396 with missing covariates were excluded, leaving 1561 for the primary analyses; 849 were included after 1:2 propensity score matching. HGB = hemoglobin, NHANES = National Health and Nutrition Examination Survey, PIR = poverty-to-income ratio, PSM = propensity score matching.

This cohort study utilized data from NHANES 1999 to 2018. Among 2265 participants with a history of stroke, 304 were excluded due to missing hemoglobin (HGB) values and 4 due to missing survival data. Of the remaining 1957 participants, 396 had incomplete information on other covariates. After exclusions, a total of 1561 participants were included in the final analysis (Fig. 1).

### 2.2. Anemia

Anemia was defined based on HGB concentration, with values <13 g/dL for men and <12 g/dL for women classified as anemic.^[22]^

### 2.3. Outcome

The primary outcomes of this study were cause-specific mortality, categorized as cardiovascular mortality, cancer mortality, and deaths from other causes. Mortality data were obtained from the National Death Index through linkage based on SEQN matching. Survival status was determined using the variable MORTSTAT, and follow-up time was defined by PERMTH_INT. Cardiovascular mortality was identified using ICD-10 codes I60 to I69, I00 to I09, I11, I13, and I20 to I51, whereas cancer mortality was defined by codes C00 to C97.

### 2.4. Variable definitions and categorization

The covariates included in this study were age, sex, race, poverty-to-income ratio (PIR), educational attainment, marital status, smoking status, alcohol consumption, diabetes mellitus (DM), hypertension, white blood cell (WBC) count, and platelet count. All data were extracted from the NHANES database and matched using the SEQN identifier. DM was defined as meeting any of the following criteria: a physician diagnosis of diabetes; use of glucose-lowering medications or insulin; a random blood glucose level >11.1 mmol/L; a 2-hour blood glucose level >11.1 mmol/L during an oral glucose tolerance test; or a HGB A1c level >6.5%. Hypertension was defined as: a physician diagnosis of hypertension; use of antihypertensive medications; or an average blood pressure ≥140/90 mm Hg. Smoking status was classified as “Never” (fewer than 100 cigarettes smoked in a lifetime), “Former” (more than 100 cigarettes smoked but not currently smoking), and “Current” (more than 100 cigarettes smoked and currently smoking). Alcohol consumption was categorized as “Never” (fewer than 12 alcoholic drinks in a lifetime), “Former” (more than 12 drinks in a lifetime but none in the past year), and “Current” (more than 12 lifetime drinks, including at least 1 drink in the past year).

## 3. Statistical analysis

All statistical analyses were performed using R version 4.4.1. Continuous variables were presented as medians with interquartile range, and categorical variables were summarized as frequencies and percentages. The nonlinear association between HGB and all-cause mortality was evaluated using restricted cubic spline (RCS) analysis. The association between anemia and cause-specific mortality was assessed using a competing risks regression model. In this model, the target cause of death was coded as 1, other causes of death as 2, and survival as 0. Subdistribution hazard ratio (sHR) and 95% confidence interval (CI) were reported to quantify the associations. cumulative incidence functions (CIFs) were used to visualize the risk of death over time. To mitigate confounding in the CIF analyses, we applied propensity score matching (PSM) using a 1:2 nearest-neighbor algorithm to preserve statistical power while improving covariate balance. The propensity score included age, sex, race, PIR, educational attainment, marital status, smoking status, alcohol consumption, DM, and hypertension. The competing risks model was adjusted for key covariates, including age, sex, race, PIR, education level, marital status, smoking status, alcohol consumption, DM, and hypertension. A two-sided *P* value < .05 was considered statistically significant.

## 4. Results

### 4.1. 1. Baseline characteristics and study outcomes

Table 1 presents the baseline characteristics of the study population. A total of 1561 participants with a history of stroke were included, with a median age of 69 years; 770 (49.3%) were female. The median follow-up duration was 79 months, during which 735 participants died. Among the cohort, 1278 participants did not have anemia, while 283 met criteria for anemia. Significant differences were observed between the anemic and non-anemic groups in several baseline characteristics, including age, race, smoke, drink, HGB, WBC, hypertension, DM, survival status, and follow-up duration (all *P* < .05). Specifically, participants with anemia were older, had a lower proportion of White individuals, were less likely to smoke or consume alcohol, had lower HGB and WBC levels, a higher prevalence of hypertension and DM, a greater proportion of deaths, and a shorter follow-up period. There were no significant differences between the 2 groups in other baseline variables, including cause-specific mortality (Table 1).

**Table 1:** Baseline characteristics and survival data of study participants.

	All, n = 1561	No anemia, n = 1278	Anemia, n = 283	*P*
Age (yr)	69.0 (59.0; 78.0)	68.0 (58.0; 77.8)	74.0 (65.0; 80.0)	<.001
Female	770 (49.3%)	643 (50.3%)	127 (44.9%)	.112
Race				
White	840 (53.8%)	714 (55.9%)	126 (44.5%)	<.001
Black	373 (23.9%)	262 (20.5%)	111 (39.2%)	
Mexican American	176 (11.3%)	151 (11.8%)	25 (8.83%)	
Other	172 (11.0%)	151 (11.8%)	21 (7.42%)	
PIR	1.63 (1.02;2.86)	1.58 (1.00; 2.88)	1.77 (1.08; 2.70)	.475
Education				
<High school	577 (37.0%)	473 (37.0%)	104 (36.7%)	.988
>High school	584 (37.4%)	477 (37.3%)	107 (37.8%)	
High school	400 (25.6%)	328 (25.7%)	72 (25.4%)	
Marital				
Married	764 (48.9%)	622 (48.7%)	142 (50.2%)	.659
Never married	107 (6.85%)	91 (7.12%)	16 (5.65%)	
Separated	690 (44.2%)	565 (44.2%)	125 (44.2%)	
Smoke				
Never	632 (40.5%)	515 (40.3%)	117 (41.3%)	<.001
Former	582 (37.3%)	455 (35.6%)	127 (44.9%)	
Current	347 (22.2%)	308 (24.1%)	39 (13.8%)	
Drink				
Never	268 (17.2%)	229 (17.9%)	39 (13.8%)	<.001
Former	569 (36.5%)	434 (34.0%)	135 (47.7%)	
Current	724 (46.4%)	615 (48.1%)	109 (38.5%)	
HGB (g/dL)	13.8 (12.7; 14.8)	14.1 (13.3; 15.0)	11.6 (11.1; 12.2)	<.001
WBC (10^9^/L)	7.10 (5.80; 8.60)	7.30 (5.90; 8.80)	6.50 (5.30; 8.10)	<.001
Platelet (10^9^/L)	233 (195; 278)	234 (197; 278)	225 (184; 284)	.111
Hypertension	1262 (80.8%)	1014 (79.3%)	248 (87.6%)	.002
Diabetes	606 (38.8%)	465 (36.4%)	141 (49.8%)	<.001
Assumed dead	735 (47.1%)	569 (44.5%)	166 (58.7%)	<.001
Cause of death				
Cardiovascular	299 (40.7%)	231 (40.6%)	68 (41.1%)	.752
Cancer	114 (15.5%)	90 (15.8%)	24 (14.5%)	
Other	322 (43.8%)	248 (43.6%)	74 (44.6%)	
Follow-up time (mo)	79.0 (41.0; 124.0)	84.0 (45.0; 131.0)	59.0 (28.5; 96.0)	<.001

Data are presented as median (Q1; Q3) or n (%).

HGB = hemoglobin, PIR = poverty-to-income ratio, Q1 = 1st quartile, Q3 = 3rd quartile, WBC = white blood cell.

### 4.2. 2. Association between anemia and cause-specific mortality

**Figure 2.**
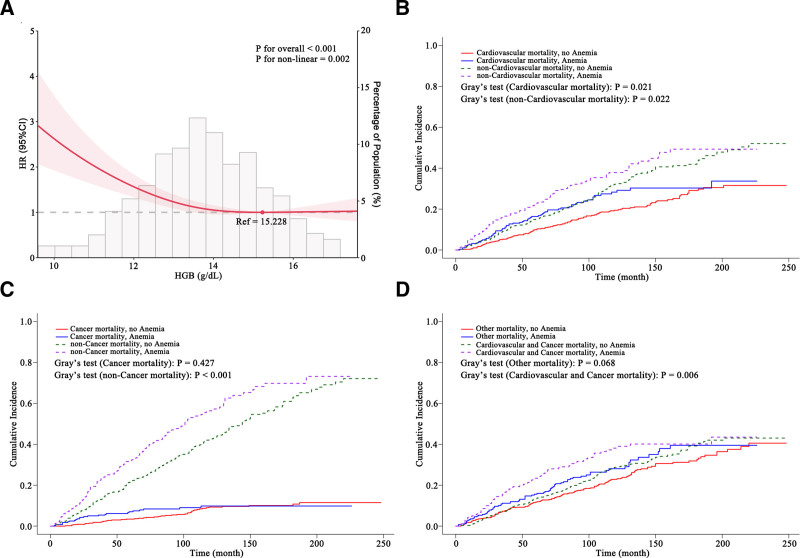
Association between anemia and cause-specific mortality. (A) Cox model with restricted cubic splines for HGB and all-cause mortality, adjusted for age, sex, race, poverty-to-income ratio, education, marital, smoke, drink, diabetes, and hypertension; knots at the 10th/50th/90th percentiles; reference HGB = 15.23 g/dL; shaded area = 95% CI; *P* values (overall and non-linear) from Wald tests are shown. (B–D) Cumulative incidence functions from a Fine–Gray competing-risk framework in the 1:2 PSM cohort, comparing anemic vs non-anemic participants for: cardiovascular vs non-cardiovascular deaths (B), cancer vs non-cancer deaths (C), and other causes vs cardiovascular + cancer deaths (D). Gray’s test *P*-values are displayed. CIF = cumulative incidence function, HGB = hemoglobin, PSM = propensity score matching.

RCS analysis revealed a nonlinear association between HGB levels and all-cause mortality, with a markedly increased risk observed when HGB fell below approximately 14 g/dL (Fig. 2A). To minimize confounding, PSM was performed to balance age, sex, race, PIR, education, marital, smoke, drink, hypertension, and DM between anemic and non-anemic participants. After matching, baseline characteristics were well balanced between the 2 groups (Table 2; Figs. S1A–D and S2, Supplemental Digital Content, https://links.lww.com/MD/Q362).

**Table 2:** Baseline characteristics and survival data of study participants after propensity score matching.

	All, n = 849	No anemia, n = 566	Anemia, n = 283	*P*
Age (yr)	73.0 (65.0; 80.0)	73.0 (65.0; 80.0)	74.0 (65.0; 80.0)	.798
Female	384 (45.2%)	257 (45.4%)	127 (44.9%)	.942
Race				
White	404 (47.6%)	278 (49.1%)	126 (44.5%)	.380
Black	299 (35.2%)	188 (33.2%)	111 (39.2%)	
Mexican American	82 (9.66%)	57 (10.1%)	25 (8.83%)	
Other	64 (7.54%)	43 (7.60%)	21 (7.42%)	
PIR	1.65 (1.06; 2.78)	1.58 (1.03; 2.79)	1.77 (1.08; 2.70)	.533
Education				
<High school	320 (37.7%)	216 (38.2%)	104 (36.7%)	.908
>High school	319 (37.6%)	212 (37.5%)	107 (37.8%)	
High school	210 (24.7%)	138 (24.4%)	72 (25.4%)	
Marital				
Married	409 (48.2%)	267 (47.2%)	142 (50.2%)	.679
Never married	53 (6.24%)	37 (6.54%)	16 (5.65%)	
Separated	387 (45.6%)	262 (46.3%)	125 (44.2%)	
Smoke				
Never	356 (41.9%)	239 (42.2%)	117 (41.3%)	.966
Former	376 (44.3%)	249 (44.0%)	127 (44.9%)	
Current	117 (13.8%)	78 (13.8%)	39 (13.8%)	
Drink				
Never	111 (13.1%)	72 (12.7%)	39 (13.8%)	.830
Former	400 (47.1%)	265 (46.8%)	135 (47.7%)	
Current	338 (39.8%)	229 (40.5%)	109 (38.5%)	
HGB (g/dL)	13.2 (12.1; 14.4)	13.9 (13.2; 14.9)	11.6 (11.1; 12.2)	<.001
WBC (10^9^/L)	6.90 (5.60; 8.40)	7.05 (5.90; 8.50)	6.50 (5.30; 8.10)	<.001
Platelet (10^9^/L)	226 (190;271)	226 (192; 268)	225 (184; 284)	.975
Hypertension	742 (87.4%)	494 (87.3%)	248 (87.6%)	.971
Diabetes	422 (49.7%)	281 (49.6%)	141 (49.8%)	.986
Assumed dead	464 (54.7%)	298 (52.7%)	166 (58.7%)	.113
Cause of death				
Cardiovascular	182 (39.2%)	114 (38.3%)	68 (41.1%)	.582
Cancer	69 (14.9%)	45 (15.1%)	24 (14.5%)	
Other	213 (45.9%)	139 (46.6%)	74 (44.6%)	
Follow-up time (mo)	73.0 (38.0; 116.0)	83.0 (42.0; 128.0)	59.0 (28.5; 96.0)	<.001

Data are presented as median (Q1; Q3) or n (%).

HGB = hemoglobin, PIR = poverty-to-income ratio, Q1 = 1st quartile, Q3 = 3rd quartile, WBC = white blood cell.

CIF curves based on the matched cohort demonstrated that anemia was significantly associated with increased cardiovascular mortality and non-cardiovascular mortality (Fig. 2B). Anemia was not significantly associated with cancer mortality but was associated with an increased risk of non-cancer mortality (Fig. 2C). Similarly, anemia was significantly associated with the composite of cardiovascular and cancer deaths, but not with deaths from other causes (Fig. 2D). In the competing risks regression model, anemia was significantly associated with an increased risk of cardiovascular mortality (sHR, 1.40; 95% CI: 1.02–1.90; *P* = .03), but showed no significant association with cancer mortality or mortality from other causes (Table 3).

**Table 3:** Association between anemia and cause-specific mortality among stroke survivors: competing risks regression model.

	sHR	95% CI	*P*
Cardiovascular mortality	1.40	(1.02, 1.90)	.03
Cancer mortality	1.16	(0.71, 1.89)	.57
Other-cause mortality	1.22	(0.91, 1.64)	.19

Models were adjusted for age, sex, race, poverty-income ratio, education, marital, smoke, drink, diabetes, and hypertension.

CI = confidence interval, sHR = subdistribution hazard ratio.

## 5. Discussion

This study used NHANES 1999 to 2018 data to examine a large cohort of stroke survivors. Baseline confounders were controlled through PSM, and cause-specific mortality was analyzed using the Fine–Gray competing risks model. The findings demonstrated that anemia was significantly associated with an increased risk of cardiovascular mortality among stroke survivors, whereas no significant association was observed with cancer-related or other-cause mortality. While most prior studies have focused on the relationship between anemia and all-cause mortality, this study systematically evaluated the association between anemia and specific causes of death, thereby extending the existing body of evidence related to post-stroke risk stratification. These results suggest that anemia may be an independent risk factor for cardiovascular mortality in stroke survivors and underscore the importance of routinely assessing and managing anemia in this population.

Several studies have investigated the association between anemia and stroke prognosis, encompassing various subtypes of stroke and focusing primarily on clinical outcomes such as functional recovery and mortality. In patients with ischemic stroke, anemia has been significantly associated with reduced functional independence, increased complication rates, and prolonged hospital stays.^[7]^ Similarly, in individuals with cerebral venous thrombosis, anemia was strongly associated with worse functional outcomes.^[23]^ Furthermore, anemia has been linked to poor 90-day functional prognosis regardless of whether patients received endovascular therapy, although it was not significantly associated with early neurological deficit severity.^[24,25]^ In cases of intracerebral hemorrhage, anemia has also been correlated with hematoma expansion and unfavorable clinical outcomes.^[26]^ Beyond functional outcomes, anemia has been implicated in post-stroke cognitive impairment.^[27,28]^ In terms of mortality, studies have reported that anemia is associated with an elevated risk of death among patients with cerebral venous thrombosis, with HGB levels inversely correlated with mortality.^[23]^ Among stroke survivors receiving antiplatelet therapy, anemia has also been shown to significantly increase the risk of all-cause mortality.^[17]^ Although the focus of the present study differs from those of prior investigations, the findings are consistent with existing evidence, further supporting the conclusion that anemia is an important risk factor for adverse outcomes in patients with stroke.

Several distinctions exist between the present study and prior research. Notably, this study featured a longer median follow-up duration of 79 months and focused on cause-specific mortality, including cardiovascular, cancer-related, and other causes of mortality. The use of PSM and competing risks regression modeling represents a methodological strength and contributes to the novelty of this analysis. Additionally, the effect of anemia on cardiovascular mortality appeared to be time-dependent. As shown in Figure 2B, the increased risk associated with anemia was more pronounced within the first 150 months of follow-up and began to diminish after approximately 200 months. Although the association between anemia and cause-specific mortality appeared attenuated in Tables 1 and 2, the shorter follow-up time observed in the anemic group suggests a potential survival disadvantage, which aligns with the findings from the CIF curves (Tables 1 and 2; Fig. 2B–D). Furthermore, while the associations between anemia and cancer mortality or other-cause mortality were not statistically significant in the primary analysis, this may be attributable to limited statistical power due to small event counts. When cancer mortality and non-cardiovascular mortality were combined for analysis, the association with anemia reached statistical significance (Fig. 2B), warranting cautious interpretation. Future research is needed to further elucidate the impact of anemia on stroke prognosis. This includes investigating the role of different anemia subtypes, evaluating the potential benefits of anemia-targeted interventions, and exploring more granular cause-of-death classifications in post-stroke populations.

Several mechanisms may underlie the association between anemia and increased mortality risk in patients with stroke. In ischemic stroke, reduced oxygen-carrying capacity due to low HGB levels may exacerbate hypoxia in the ischemic penumbra.^[29]^ Anemia-induced hyperdynamic circulation can promote thrombus dislodgement and trigger vascular endothelial inflammation.^[30]^ Additionally, anemia is often associated with chronic inflammation and comorbidities – such as chronic kidney disease and malnutrition – that may increase stroke susceptibility and worsen outcomes.^[31,32]^ In the context of intracerebral hemorrhage, anemia may aggravate hypoxia in the metabolic penumbra and facilitate hematoma expansion, thereby contributing to poorer prognosis and elevated mortality risk.^[33–36]^ Anemia also imposes significant physiological stress on the cardiovascular system. Insufficient oxygen delivery can directly impair cardiomyocyte function, leading to decreased ATP production and increased oxidative stress and cellular damage.^[37–40]^ Chronic hypoxia may induce apoptosis and interstitial fibrosis, ultimately resulting in progressive structural and functional deterioration of the myocardium.^[39,41]^ To compensate for reduced oxygen transport, anemia causes an increase in heart rate and cardiac output, which, when sustained, places a substantial mechanical burden on the heart.^[42]^ Moreover, anemia activates the sympathetic nervous system and the renin-angiotensin system, and is associated with elevated levels of pro-inflammatory cytokines, further contributing to myocardial injury.^[43,44]^ Taken together, these mechanisms suggest that anemia may increase overall mortality in stroke survivors through both cerebrovascular and cardiovascular pathways, with particularly strong implications for cardiovascular mortality.

Several mechanisms may account for the higher prevalence of anemia among individuals with a history of stroke. First, anemia and stroke share common antecedents – including hypertension, diabetes, and chronic kidney disease – that cluster in this population.^[45–48]^ Second, anemia is a risk factor for incident stroke; thus, individuals with anemia are overrepresented among stroke survivors.^[49,50]^ Third, poststroke dysphagia, hemiparesis, and functional dependence can reduce nutritional intake and thereby contribute to anemia.^[51,52]^ Fourth, long-term antithrombotic therapy (antiplatelet agents and anticoagulants) increases gastrointestinal and occult bleeding, thereby predisposing to anemia.^[53–55]^ Fifth, recurrent hospitalizations expose patients to frequent phlebotomy and fluid administration or transfusion, which can cause iatrogenic anemia and hemodilution.^[56,57]^ Finally, stroke induces systemic inflammation, which contributes to anemia of inflammation through disordered iron homeostasis.^[58–60]^ Collectively, these factors likely underlie the elevated prevalence of anemia after both ischemic and hemorrhagic stroke.

This study has several limitations. First, as a retrospective cohort study, it is subject to inherent limitations in establishing causal relationships. Although adjustments were made for multiple confounding variables, residual confounding from unmeasured factors cannot be excluded. Second, information on the type and etiology of anemia was not available, preventing further stratified analyses. Third, detailed clinical data on stroke subtype and severity were lacking, which may have influenced prognosis and mortality risk. Fourth, we did not exclude participants with coexisting conditions common after stroke (e.g., hypertension, diabetes, chronic kidney disease, malignancy, or cardiovascular disease). This approach reflects real-world practice but may introduce confounding by indication and disease severity despite adjustment. Finally, HGB levels were measured only at baseline, and changes in HGB over time could not be assessed.

## 6. Conclusion

Based on data from NHANES, this study found that anemia was significantly associated with an increased risk of cardiovascular mortality among stroke survivors, but not with cancer-related or other-cause mortality. These findings indicate that anemia may be an important risk factor for cardiovascular death after stroke, highlighting the need for greater clinical attention to anemia in post-stroke care. Further research is warranted to assess whether correcting anemia can improve cause-specific outcomes in this population.

## Acknowledgements

The authors have no acknowledgments to declare.

## Author contributions

**Conceptualization:** Jianya Chen, Xiaoping Xie, Hui Mai, Qiufei Cheng.

**Data curation:** Jianya Chen, Xiaoping Xie.

**Formal analysis:** Jianya Chen.

**Funding acquisition:** Hui Mai.

**Investigation:** Jianya Chen.

**Methodology:** Jianya Chen, Xiaoping Xie.

**Project administration:** Qiufei Cheng.

**Software:** Hui Mai.

**Supervision:** Qiufei Cheng.

**Validation:** Hui Mai, Qiufei Cheng.

**Writing – original draft:** Jianya Chen.

**Writing – review & editing:** Hui Mai.

## Supplementary Material


