# Supplementary material for: Association of anemia with cardiovascular and cancer mortality among stroke survivors: A cohort study (NHANES 1999–2018)
Source: Medicine (Baltimore). 2025 Oct 17;104(42):e45250. doi: 10.1097/MD.0000000000045250 (PMC12537180; doi:10.1097/MD.0000000000045250)

Figure S1. Distribution of Propensity Scores Before and After Matching in Anemic and Non-Anemic Groups

(A) Raw propensity score distribution in the anemia group before matching. (B) Propensity score distribution in the anemia group after 1:2 matching. (C) Raw propensity score distribution in the non-anemia group before matching. (D) Propensity score distribution in the non-anemia group after matching.


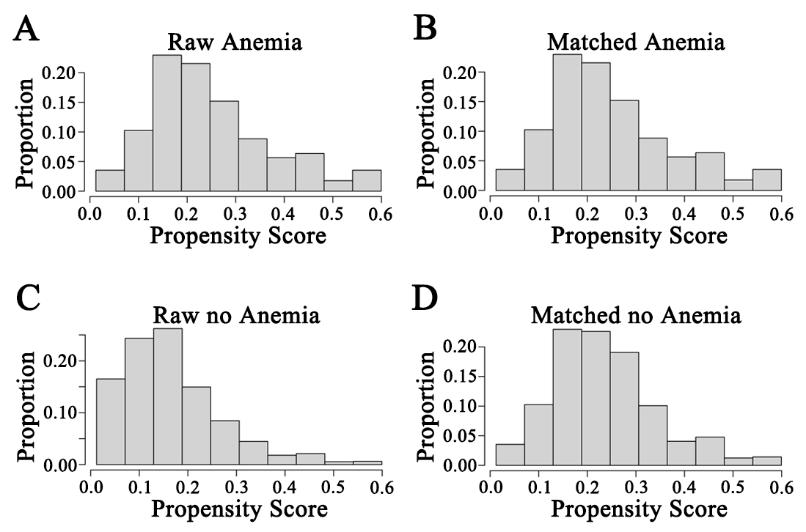


Figure S2. Absolute Standardized Mean Differences of Baseline Covariates: Pre- vs Post-Matching


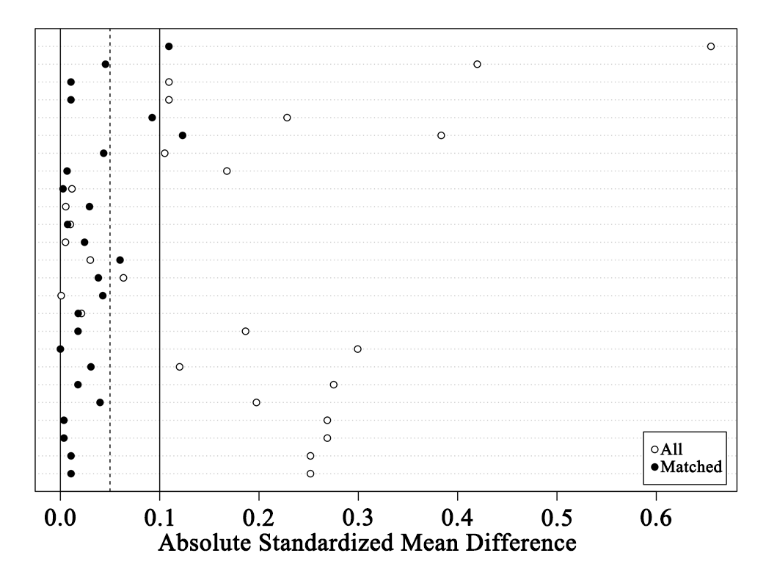

Supplement: Supplementary file 1 [file medi-104-e45250-s001.docx]
